# 

**Published:** 1864-04

**Authors:** 


### BOOKS RECEIVED.

*Announcement Harvard University*, 1864.—*Medical Department.—Summer Session, Commencing March* 14, 1864.

*The Vermont School Journal, devoted to the Educational Interests of the State. Published under the sanction of the Vermont State Teachers. Association.* Hiram Orcutt, *Editor and Proprietor, West Brattleboro.'*

*The Sanitary Commission Bulletin, Vol.* 1, *No. XI. New York, April* 1*st*, 1864.

*Report from the Hon. Josiah Quincy, jr., Hon. Alfred Hitchcock, M. D., and Horatio R. Storer, M. D., Commissioners appointed under the* 91*st Chapter of the Resolves of* 1863, *to make certain investigations of the subject of Insanity, and the disposition of persons alleged to be insane.*

---

### AMERICAN MEDICAL ASSOCIATION.

The Fifteenth Annual Meeting of the "American Medical Association" will be held in the city of New York, commencing Tuesday, June 7th, 1864, at 10 A. M.

Proprietors of Medical Journals throughout the United States and their territories are respectfully requested to insert the above notice in their issues. Guido Furman, M. D., Secretary.

New York, March, 1864.

